# Rethinking the drivers of coronavirus virulence and pathogenesis; toward an understanding of the dynamic world of mutations, indels, and recombination within the alphacoronaviruses

**DOI:** 10.1128/mbio.01921-25

**Published:** 2025-08-28

**Authors:** Ximena A. Olarte-Castillo, Laura E. Frazier, Jessica C. Gomes Noll, Annette Choi, Gary R. Whittaker

**Affiliations:** 1 Departments of Microbiology & Immunology and Public & Ecosystem Health, Cornell University 5922, Ithaca, New York, USA; Albert Einstein College of Medicine, Bronx, New York, USA

**Keywords:** *Alphacoronavirus*, feline coronavirus (FCoV), canine coronavirus (CCoV), porcine coronavirus, recombination, insertions-deletions (indel), pathogenesis, virulence, genotype

## Abstract

Alphacoronaviruses are widespread but understudied in comparison to betacoronaviruses. Within the alphacoronaviruses is the species *Alphacoronavirus-1*, which comprises distinct viruses of cats, dogs, and pigs, along with a separate species that infects mustelids—as well as other related viruses of pigs and circulating human viruses. High-pathogenicity feline coronavirus (FCoV) is infamous as the cause of feline infectious peritonitis (FIP), existing as two distinct genotypes (types 1 and 2) and transmitted as a low-pathogenicity virus. The high-pathogenicity variants arise in cats infected with FCoV, and while the mutations responsible remain enigmatic, the main determinant is the spike glycoprotein. FCoV-1 disease outcome is driven by a combination of both within- and between-host evolution. Virulence can be largely explained by the “internal mutation hypothesis,” which argues that high-pathogenicity—but poorly transmissible—variants are selected in individual cats. Canine coronaviruses are generally considered low pathogenicity but can cause severe enteritis and can be systemic. Notably, the canine coronavirus spike gene periodically recombines with FCoV-1 to generate FCoV-2, exemplified by FCoV-23, which has caused a widespread outbreak of FIP in Cyprus and has a notably truncated spike N-terminal domain (NTD). In pigs, coronaviruses often cause severe gastrointestinal disease but can become respiratory and have low pathogenicity based on what can also be considered an “internal deletion” of the spike NTD. These viruses may exist as a dynamic “metavirome” (the sum of all viral genomes present in a sample) that is in a constant state of flux, presenting notable challenges for disease surveillance and management.

## INTRODUCTION

Alphacoronaviruses are in the family *Coronaviridae*, along with three other genera; beta, gamma, and deltacoronaviruses. The *Alphacoronavirus* genus consists of viruses that infect a wide range of hosts, including humans, cats, dogs, pigs, bats, rats, and other rodents. Many of these viruses display cross-species transmission, and coinfection in a given host can lead to recombination between viral species, which drives viral evolution. Historically, alphacoronaviruses have demonstrated the ability to infect humans, and spillover events from animals to humans have occurred on several occasions, underscoring the importance of monitoring these viruses (1). For feline coronaviruses (FCoVs), a 2020 FCoV review by Jaimes et al. (2) focuses on the spike protein, as it is the main driver of coronavirus cell tropism and pathogenesis (3). In 2023, Gao et al. also published a review on the two FCoV biotypes (4). In this review, we provide an update on FCoV-1 in comparison to FCoV-2 and canine coronavirus (CCoV), including the newly emerged feline/canine recombinant virus FCoV-23 (5–8), and compare these to related viruses of pigs and mustelids and the possibilities for zoonotic or other cross-species infections.

## FELINE CORONAVIRUS (FCoV)

FCoV is one of the most important viruses of cats, affecting both domestic and wild felids. First recognized in 1963, it is now well established to be the cause of feline infectious peritonitis (FIP), which is typically lethal without therapeutic intervention (9, 10). FCoV is widespread, with the prevalence of infection in the U.S. feline population estimated at 75%–95% in multi-cat households (11), 25% in single-cat households, and approaching 100% in shelter/breeder situations. With an estimated 58 million owned cats in the United States alone (12)*,* FCoV represents a widespread endemic coronavirus that to date remains largely unexplored from a molecular evolution perspective, and with still many unanswered clinical questions.

FCoV infection can lead to three principal clinical outcomes (13): 5% of cats clear the infection without viral shedding, 70%–80% intermittently shed low levels of virus from their gastrointestinal tract, and 10%–15% have persistent high viral load shedding. This indicates that FCoV often causes persistent infections. Furthermore, FIP may occur in 5%–12% of these cases, presenting as an effusive (wet) form, with fluid accumulation, or a non-effusive (dry) form, often with neurological signs. FIP is prevalent in environments with high cat density, particularly affecting young cats under 2, and often triggered by stress (13).

### FCoV “biotypes” (phenotypes and genotypes)

FCoV has been traditionally categorized as one of two pathogenic biotypes: feline enteric coronavirus (FECV) or feline infectious peritonitis virus (FIPV). Here, we define biotype as a non-formal category—traditionally implying taxonomic connection, but applied to FCoV to imply pathogenic outcome or phenotype. Feline enteric coronavirus is the low-pathogenicity biotype, which presents with asymptomatic or mild symptoms and is proposed to infect intestinal epithelial cells based on viral shedding in the feces. Notably, the high-pathogenicity biotype, FIPV, utilizes monocytes/macrophages for systemic spread and classically presents with peritonitis in its “wet” form. The two biotypes were originally differentiated based on the “internal mutation theory,” in that a low-pathogenicity virus infects cats and then mutates (14) within an individual animal into the high-pathogenicity virus. While this basic concept has remained a mainstay of FIP pathogenesis for over a quarter century, we consider that the oversimplistic concept of an “FECV” switching to an “FIPV” is flawed, as is more recent classification of the two biotypes as “less virulent FCoV” and “FIP-associated FCoV” (15). While the pathogenesis of FIP is certainly linked to viral mutation, this is a much more complicated process than previously anticipated, and measurable disease attributes for FIP are not easily quantifiable. Early studies to assign biotypes for FCoV linked the development of FIP to two small open reading frames (ORFs), 3c and 7b. However, more recent understanding has questioned the early connection to ORFs 3 and 7 (16, 17)*,* with the emphasis for the internal mutation focusing on the spike (S) gene, principally within S2 and at the S1/S2 interface (18, 19). While defined mutations such as S:M1058L are no longer thought to be high-pathogenicity FCoV-specific—and are rather associated with systemic spread of FCoV (20)—it remains likely that the predominant genetic changes controlling viral pathogenesis lie in the S gene (21). Based on it being the principal cause of FIP, recent genomic studies linked to the biotype switch have focused on FCoV-1.

The more we uncover about the genetics of the virus, the more it is becoming apparent that the two “biotypes” are a simplified and outdated way to label the virus. We argue that the “biotype” framework is problematic because it explains FCoV infection through phenotypic presentation in order to make sense of the genotype (which is, in reality, the driver of pathogenesis). To understand the disease process, there is a need for more robust sequencing to be performed, so that we can use the genotype to successfully predict the phenotype. In this review, we intentionally use the terms “low-pathogenicity” and “high-pathogenicity” FCoV to descriptively capture the different biotypes without inference to specific markers of virulence (which remain poorly defined), and classify the two viruses as genotypes (the genetic constitution of an individual organism). It is important to note that, based on conventional taxonomic classification, all FCoVs (as well as CCoVs and certain coronaviruses of pigs) are a single virus species, *Alphacoronavirus-1* (genus *Alphacoronavirus*, subgenus *Tegacovirus*, member species *Alphacoronavirus suis*); see https://ictv.global/report/chapter/coronaviridae/coronaviridae/alphacoronavirus.

While low-pathogenicity FCoV is widely distributed and presumably highly transmissible, “FIPV” is not generally thought to be a transmissible virus—with “outbreaks” likely resulting from multiple individual low- to high-pathogenicity conversions of FCoV within a defined location. In this context, we also need to reconsider what is meant by an outbreak for FCoV, as compared to a cluster of nontransmissible viruses. There is limited molecular epidemiology of FCoV in the literature, describing what seem to be traditional horizontal transmission events (FCoV-2) and what might be better considered as clusters of distinct but closely related viral variants (FCoV-1) (22–26).

### FCoV serotypes

In addition to being a primary pathogenesis determinant, the FCoV spike is also a critical factor in antigenicity, and the virus has been traditionally considered to exist as two “serotypes” (a distinguishable feature of an organism based on an antibody response or test)—traditionally noted as serotypes I and II. Historically, the term serotype was used in the early stages of discovery for FIP, prior to the availability of robust genomic information, as it was appreciated that sera from certain cats with FIP or infected with FCoV failed to cross-react/cross-protect with sera from other cats, leading to the concept that the virus exists as two distinct serotypes. This concept was reinforced by the isolation of monoclonal antibodies to what is now known as FCoV-2, whereby distinct differences in spike protein antigenic epitopes were apparent. However, a “serotype” may in reality only reflect minor differences in defined antigenic epitopes, and there is <50% amino acid identity between the spike proteins of the two FCoVs—as such, the term serotype does not reflect their notable evolutionary differences. Within each serotype, it is apparent that antibodies produced during infection, as well as monoclonal antibodies—including those that strongly neutralize—can induce antibody-dependent enhancement of infection (ADE). This process remains poorly understood but is a major impediment to the development of an FIP vaccine (27–30).

In 2018, we presented evidence that the “serotypes” really reflect viruses representing two distinct genetic clades (a group of organisms believed to have originated from a common ancestor)—with clade A corresponding to serotype I and clade B corresponding to serotype II. This nomenclature captures the high genetic diversity that has been recognized to date, including fundamentally distinct biological properties of the two viruses (31). For example, FCoV-1 but not FCoV-2 contains a furin cleavage site (FCS) in the spike protein, which can prime the spike for virus entry. This proteolytic cleavage site in FCoV-1 may add a layer of control the virus can utilize for productive infection. In contrast, FCoV-2 results from recombination events between FCoV-1 and CCoV, where the recombinant genotype has the spike protein (and sometimes surrounding regions) from CCoV, with the rest of the genome from FCoV-1. Exchange of the spike protein through recombination results in antigenic shift.

Tables 1 and 2 summarize what is currently known about the FCoV biotypes and serotypes/genotypes.

**TABLE 1:** Comparison of low- and high-pathogenicity FCoV-1

Parameter	Low-pathogenicity FCoV	High-pathogenicity FCoV
Biotype	FECV	FIPV
ABCD guidelines	Less virulent FCoV	FIP-associated FCoV
Symptoms	Asymptomatic or mild gastrointestinal symptoms	Wet FIP—including peritonitis Dry FIP—including neurological
Cell tropism	Intestinal epithelial cells	Monocytes and macrophages
Transmissible	Yes	No

**TABLE 2:** Comparison of FCoV-1 and FCoV-2

Parameter	FCoV-1	FCoV-2
Serotype	Serotype I	Serotype II
Clade	Clade A	Clade B
Clinical prevalence	>90%	<10%
Spike protein origin	FCoV	CCoV
Cell culture	No	Yes
Receptor	Unknown	APN
S1/S2 cleavage site or FCS	Yes	No
S2´ cleavage site	Yes	Yes

### FCoV methodology differences

Aside from serological studies, it is important to note that most current diagnostic testing methodologies (either immunohistochemistry [IHC] or PCR) do not discriminate between FCoV-1 or FCoV-2. FCoV-1 has a lot of clinical sequencing data, but very little *in vitro* data. Alternatively, FCoV-2 has little clinical sequencing data but many *in vitro* studies. The differences between the approaches with which FCoV-1 and FCoV-2 have been studied illustrate how both surveillance of ongoing infections and basic science work need to be done in parallel. Sequencing of circulating viruses validates and reinforces results from basic science or can provide insight into the genetic adaptations viruses gain when being propagated in cell culture. FCoV-2 is well understood from a basic science perspective because it is possible to grow and propagate in cell culture. However, because not much sequence data has been obtained from clinical FCoV-2 variants, it has not been possible to compare circulating viruses to the lab-adapted strains that have been highly studied.

### FCoV cellular tropism

Despite many years of study, cell tropism of FCoV across the FECV/FIPV spectrum, and of CCoV, remains an open question. In part, this is because cell culture-based studies can easily lead to misappropriation of viral tropism. Coronaviruses can select tropism variants extremely easily, with a “hot-spot” of selection in the spike cleavage sites; for examples, see references 32, 33. While rapid cell culture adaptation has been known for many years, the notable loss of the “furin cleavage site” of the prototype severe acute respiratory syndrome coronavirus 2 (SARS-CoV-2) isolate WA-1 in VeroE6 cells readily illustrated this process to the wider scientific community. This occurred mainly through indels, but also through point mutations (see references 34, 35 for examples). Related to this, the passage history of FCoV-2 FECV-1683 included up to four passes in CRFK and/or fcwf-4 cells prior to the apathogenic phenotype documented upon experimental challenge of cats (36), and sequences of the original isolate are not available. FCoV-1 is almost impossible to isolate in conventional cell culture, except for the highly cell-adapted FCoV-Black virus. It has notably mutated spike cleavage sites and has likely also picked up heparin-sulfate binding activity (C. Menchaca and G. R. Whittaker, unpublished data). While both FCoV-2 and CCoV-2 are readily isolatable, and with a well-characterized receptor, aminopeptidase N (APN), a specific molecular receptor for FCoV-1 remains unidentified to date. FCoV-1 and FCoV-2 are also able to recognize Fc receptors *in vivo* (30, 37), driving ADE for FIP. Clinically, current “gold standard,” antibody-based IHC approaches are limited with respect to identification of specific cell types *in vivo*. RNA-based *in situ* hybridization approaches are much better (38), but not commonly used.

Despite many years of study, the enteric tropism of FCoV remains unclear. While some clinical studies appear to show robust infection of epithelial cells (6, 39), an experimental study of low-pathogenicity FCoV-1 showed that the epithelial cells in the colon had pathology, but they were barely infected, especially compared to the lymph node macrophages (40). For many FCoV infections, pathology may not be linked to the clinical signs.

### The FCoV-1 furin cleavage site (S1/S2) disruption hypothesis

FCoV-1 accounts for most coronavirus infections of cats—and cases of FIP. It is the most-studied virus in the species *Alphacoronavirus-1* from a clinical perspective. FCoV-1 cannot be readily isolated, making it difficult to study *in vitro*. Therefore, robust sequencing of clinical FCoV-1 cases has been the principal means of advancing the study of this virus.

The progression of low-pathogenicity to high-pathogenicity FCoV has been linked to several “hotspot” genomic changes, including in the 7b, 3c and S genes. While multiple genomic changes likely account for ultimate conversion to FIP, mutations at the FCS are strongly linked to high-pathogenicity FCoV-1 (18, 19, 23, 41–44). The FCS in FCoV-1 is a structural loop located close to the interface of the spike S1 (receptor binding domain) and S2 (fusion domain) subdomains. Furin is a ubiquitous cellular protease that minimally recognizes two critical arginine (R) residues, accompanied by a complex series of additional amino acids, often with a preference for additional basic residues and serine (S) residues that promote docking of the cleavage loop substrate into the enzyme binding site (45–47). Due to its location in spike, this region is often known as the “S1/S2 cleavage site”—although it is actually located approx. 70 aa within spike domain D, rather than at the expected interface of the S1 and S2 subdomains (48). In circulating low-pathogenicity FCoV-1, the S1/S2 cleavage site contains a strong consensus motif for cleavage activation by furin. While specific mechanistic information remains elusive, by analogy to the more studied betacoronaviruses (where a furin cleavage site can be common), proteolytic cleavage at this site is likely to alter cell tropism and entry pathways, and possibly drive virus transmission, promote membrane fusion, and affect spike stability (49). This review is the first time the mutations at the FCoV-1 furin cleavage site are referred to as a “disruption” hypothesis.

In 2013, we showed that amino acid sequence changes in the furin cleavage site of FCoV that decreased cleavage are highly correlated with conversion to FIP (18). Our initial molecular analysis of the S1/S2 cleavage site identified a consensus sequence in low-pathogenicity FCoV samples of (S/T/Q)-R-R-(S/A)-R-R-S in 30 fecal samples from apparently healthy cats (i.e., “FECV, or low-pathogenicity FCoV”), and a disruption of this motif in 22 tissue samples from cats clinically confirmed to have FIP based on IHC analysis. In this initial pilot study, the disruption of the consensus cleavage motif was present in 100% of FIP cats—although not in all tissues. The result of FIP-positive cats having 100% mutated and apparently nonfunctional FCS led to the hypothesis that “uncleaved” spikes are somehow functionally responsible for the “FIPV, or high-pathogenicity FCoV” biotype. Disruption of the FCS was shown to decrease proteolytic cleavage. Peptide cleavage assays show this effect *in vitro*, but for FCoV-1, this has not been studied in live virus or *in vivo*. Subsequent case studies of individual cats and follow-up of a localized FIP outbreak in an animal shelter also confirmed the 100% correlation between mutated/non-functional FCS and high-pathogenicity FCoV (23, 43, 44). Independent validation of S1/S2 mutations as drivers of FIP has been limited, in part due to technical difficulties reported by others in sequencing this region of spike—although recent epidemiology studies from China have provided some genomic support for this hypothesis (41). Sequence analysis of the spike gene from a series of FIP cats in clinical trials testing antiviral drugs has also shown that the majority of S1/S2 sites were disrupted (42).

Notably, in 2023, a decade after our initial pilot study was published, an unbiased genomic analysis has provided additional support for the “FCS disruption hypothesis,” which identified the FCoV-1 S1/S2 loop as a main genomic region that evolves under different selective regimes between high- and low-pathogenicity FCoVs (21). This work also identified evidence of selection pressure acting on site “1058” (M1058L)—but not on ORFs 3c or 7b. “M1058L” has long been attributed to systemic spread of FCoV (but not with FIP *per se*), and we now hypothesize that this mutation acts to stabilize the spike protein and to offset the functional traits imparted by subsequent FCS (and other) mutations. FCoV-1 spike also contains a second cleavage-activation site (S2′) that is also mutated in many FIP cases (19), but, as with FCoV-2, it remains poorly understood from a functional perspective.

Overall, for FCoV-1, we argue that pathogenic variants mainly derive from accumulated point mutations, with some recent evidence for insertions-deletions (indels [50]). The point mutations/indels appear to be mainly present in certain “hot spots” within the spike, including the spike protein cleavage sites, “site 1058,” and in the N-terminal domain (NTD).

### FCoV quasispecies

An understanding of FCoV-1 links to the general concept that for RNA viruses, pathogenesis is part of quasispecies diversity (51). Over the years, this concept has been exploited to great effect in studies of human immunodeficiency virus (HIV-1) (52) and hepatitis C virus (HCV) (53) and, most recently, for SARS-CoV-2 (54). For HIV and HCV, it is well established that these viruses cause chronic or persistent infections in specific tissues, with the virus also present in specific “latent” compartments without productive replication. The presence of the virus in such compartments plays a major role in the efficacy of antiviral drugs (which can only target the actively replicating compartment). “Sanctuary” compartments can also be established where the virus is protected from the immune response or antiviral drugs due to strong barriers between this site and other anatomical compartments, such as the central nervous system (55). Increasing evidence suggests that coronavirus infections can also take advantage of such persistent or sanctuary sites. Future studies of FCoV-1 represent a new way to merge population dynamics and phylogenetics to understand disease outcomes, as it has widespread tissue distribution linked to its pathogenesis—i.e., to set a novel precedent in a discipline that has been termed “phyloanatomy” (56–58).

### FCoV antivirals

FIP has recently lost its reputation as an invariably lethal infection due to the availability of antiviral drugs originally developed for coronavirus disease 2019 (COVID-19) and other viral diseases of humans, including hepatitis C and Ebola. There are now three basic therapeutic classes that are being used in differing ways in different countries based on the availability of approved or non-approved drugs through regulatory agencies—and with highly variable clinical management and use of molecular diagnostics. The three drug classes are nucleoside analogs (GS441524/remdesivir), protease inhibitors (GC376/paxlovid), and mutagens (molnupiravir/EIDD-2801).

The understanding of FCoV-1 infection as a quasispecies is essential for an understanding and clinical management of the antiviral drugs (such as remdesivir/GS441524 and molnupiravir), which are repurposed COVID drugs rapidly coming into widespread use for treatment of FIP in cats (see references 59–62 for examples). Without further evaluation of possible sanctuary sites and ability of viruses to be shed following treatment, treated animals may be better defined as “in remission,” rather than “cured.” Based on its mechanism of action as a mutagen that accelerates virus evolution (60, 63, 64)*,* molnupiravir may be especially problematic for FCoV-1-type infections—with viral dynamics being highly adaptive processes (65, 66). However, the development of traditional antiviral resistance may technically be more of a problem with GS441524-like compounds, based on their widespread use and mechanism of action as a nucleoside analog (61). Current clinical trials are also investigating the use of paxlovid as an FIP treatment.

### FCoV-2 pathogenesis

FCoV-2 is a recombinant of FCoV-1, in which a region of the genome—including the spike gene—is obtained from CCoV-2. Since the S genes of FCoV-1 and FCoV-2 (CCoV-2) are highly divergent (<50% amino acid identity), virus-host interactions like cell entry and tropism, antigenicity, and host range are vastly different for the two viruses. For example, in cell culture, FCoV-2 grows readily, while FCoV-1 does not. For this reason, the mechanisms of cell entry of FCoV-2, including the molecular interaction with its host cell receptor, APN (also known as CD13), are well known (67).

**Fig 1:**
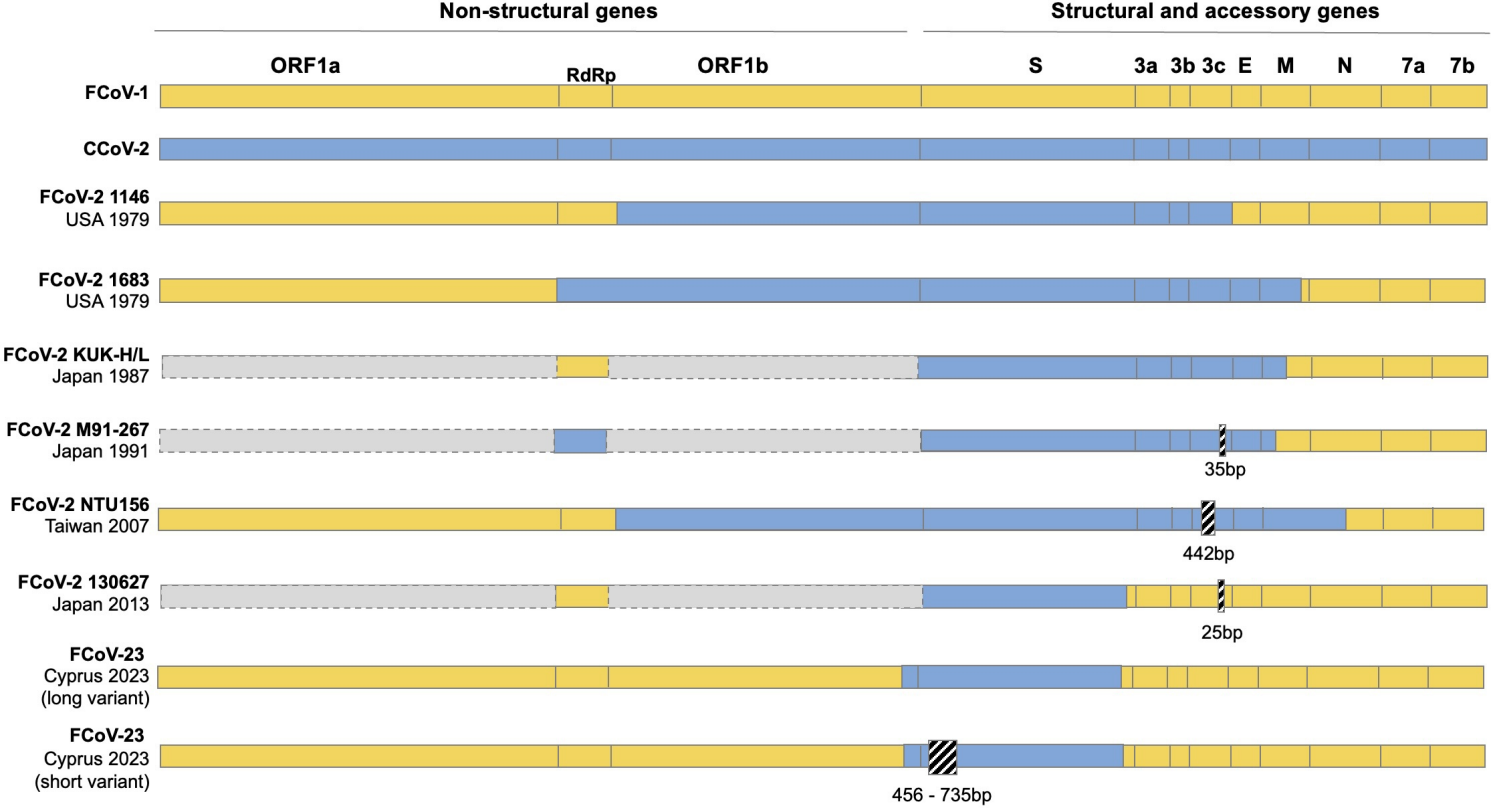
Graphical depiction of the genomic diversity of FCoV-2 and FCoV-23 recombinant variants reported to date. The position of each ORF or gene along the genome is shown at the top. Within ORF1b, the RNA-dependent RNA polymerase (RdRp) position is also shown. The genome of FCoV-1 is in yellow, and the one of CCoV-2 is in blue. FCoV-2 is a recombinant between FCoV-1 (yellow) and CCoV-2 (blue). In white are regions of the genome from which the sequence has not yet been obtained. A black box with diagonal white lines indicates deletions. The deletion size is indicated below the box in base pairs (bp). Each variant is named to the left of its respective genome. The FCoV-2 variants used (GenBank accession numbers) are 1146 (AY994055), 1683 (JN634064), KUK-H/L (AB781789), M91-267 (AB781788), NTU156 (GQ152141), 130627 (AB907624), FCoV-23 short variants (PQ133176, PQ133177, PQ133179, PQ133181, PQ133184, PQ133185, PQ133188, PQ133190, PQ133194), and FCoV-23 long variant (PQ133182).

In contrast to FCoV-1, much less is known about the genetic diversity of FCoV-2 circulating in domestic cats, with relatively few sequences available. Comparative genetic studies have revealed different FCoV-2 variants with different recombination breaking points along the genome (Fig. 1), which indicates that recombination between FCoV-1 and CCoV-2 has occurred on multiple occasions. Genetic identification of FCoV-2 has been done mostly targeting a region in the 5′-end of the S gene (68). Although this assay is sufficient to detect FCoV-2, differentiate it from FCoV-1, and detect coinfections, sequencing the complete genome is essential to detect different recombinant variants and to identify their origin. Likewise, sequencing a small region of S does not differentiate between FCoV-2 and CCoV-2. Differentiating whether cats are infected with FCoV-2 or CCoV-2 is also essential to understand if they act as mixing vessels for the recombination between FCoV-1 and CCoV-2. *In vitro* assays suggest that APN of the domestic cat allows entry of CCoV-2, FCoV-2, and transmissible gastroenteritis virus (TGEV [69]). However, compared to FCoV-1, FCoV-2 is much less prevalent in domestic cats (68, 70, 71), typically considered to be <10% of FCoV-infected animals. FCoV-2 was reported in the feces of healthy and diseased cats, and in the pleural and abdominal fluids and tissues of diseased cats. Coinfections can occur—but seem to be rare. Although early experiments showed that different variants of FCoV-2 may be more virulent than others (36), whether FCoV-2 can be differentiated into FECV/low-pathogenicity and FIPV/high-pathogenicity is less clear. Notably, the virus, typically referred to as FECV-1683, was originally isolated from a cat that had severe clinical signs, including infection of lymphoid tissue.

Studies of FCoV-2 have disproportionally focused on cases of FIP, and assessment of natural infection by CCoV-2 in cats is limited to an early study of CCoV infection in FIP cats (72), along with a recent study of a series of captive snow leopards (*Panthera uncia*) with CCoV infection linked to severe gastroenteritis (73). It remains unclear how commonly cats are infected with CCoV and in what species the recombination event takes place. While the reported recombinant genomes (Fig. 1) share the common feature of recombinant regions that include distinct spike genes, recombination including other parts of the genome is likely, including ORF3 which is unique to CCoV-1 (see below).

### The S2′ cleavage site

Compared to FCoV-1, and as expected for an *Alphacoronavirus*, there is only a single readily identifiable cleavage site in the spike protein of FCoV-2 (S2′). The S2′ cleavage sites of FCoV-1 and FCoV-2 have notably different consensus sequences (KR|S for FCoV-1 and RKYR|S for FCoV-2). These consensus sequences are highly conserved, except for a few notable substitutions that have been reported. An R-G substitution at the expected S2′ cleavage position in a few FCoV-2 variants has been reported. This may affect cell tropism (74), but not necessarily virulence. This R-G substitution has also been observed in porcine epidemic diarrhea virus (PEDV) (an *Alphacoronavirus* in the subgenus *Pedacovirus*) and may be a cell culture adaptation (75). The same substitution also occurs with ferret CoVs, but its relationship to disease outcome in this case also remains unclear (76). Additionally, a small minority of FCoV-1 isolates contain an R-S substitution at S2′ (i.e. UU47, as well as viruses in FIP cats identified in Licitra et al. [19]).

## CORONAVIRUSES OF FERRETS AND MINK

Mustelids such as mink and ferrets are susceptible to a range of both human and species-specific coronaviruses (77, 78), and several species-specific enteric alphacoronaviruses have been described, including ferret enteric coronavirus (FRECV), ferret systemic coronavirus (FRSCV), and epizootic catarrhal gastroenteritis in mink (79–81). While coronavirus infections in animals are common, a highly pathogenic FIP-like disease presentation is not—and ferrets seem to be the only species beyond cats that present with the prototypical granulomatous lesions seen with FIP (as described for FRSCV). However, relationships between pathogenic outcome and individual viruses are not clear in ferrets; e.g., we reported on systemic disease, including bone marrow involvement, in an animal infected with a virus closely related to the presumed enteric virus FRECV-MSU-2 based on the spike sequence. Together, ferret and mink enteric coronaviruses comprise a distinct species, previously proposed as *Alphacoronavirus-2,* though not currently in use by the International Committee on Taxonomy of Viruses (ICTV). Instead, mink coronavirus 1 is currently classified by ICTV as subgenus *Minacovirus*, along with ferret coronavirus. While not studied extensively, recombination may be frequent in these viruses. In two strains reported by Minami et al., phylogenetic analysis has indicated potential recombination events leading to the emergence of two additional strains, Saitama-1 and Aichi-1 (82). Lamers and colleagues have similarly shown recombination events among the S, 3c, and E genes of the FRCoVs through comparison of a strain identified in the Netherlands (designated FRCoV-NL-2010) to the previously described FRSCV MSU-1 and FRECV MSU-2 (83). An R-G substitution at the expected S2′ cleavage position (as with FCoV-2 and PEDV) in some ferret coronaviruses variants has also been reported; however, it is currently unclear how this is related to viral pathogenesis.

## CANINE CORONAVIRUS (CCoV)

CCoV is a well-established enteric pathogen of dogs—hence its alternative name canine enteric coronavirus (CECoV) (9, 10), which differentiates it from canine respiratory coronavirus (CRCoV); CCoV/CECoV, like FCoV and TGEV, lies within the *Alphacoronavirus-1* species, whereas CRCoV is distinct and is a betacoronavirus (embecovirus; species *Betacoronavirus-1*), closely related to bovine coronavirus (84, 85). As with FCoV, CCoV exists as two serotypes or types (clades), CCoV-1 and CCoV-2, with CCoV-2 being the predominant circulating form (or the one targeted for surveillance).

CCoV-2 was first isolated in 1971 and has since been found in what appears to be three distinct subtypes. Originally classified as CCoV-Ia and CCoV-IIa (here termed CCoV- and CCoV-2b), these subtypes have been well documented and are differentiated by having distinct NTD in their spike. CCoV-2b is the result of a recombination event with a TGEV-like virus; thus, it can be deduced that the CCoV-2a NTD is of canine origin, and the CCoV-2b NTD is of porcine origin. There also exist CCoVs with a third distinct NTD closely related to CCoV-1, which is in itself evolutionarily linked to FCoV-1; such viruses have been referred to as CCoV-IIc (CCoV-2c) (86, 87), with other examples of recombinant viruses possibly spanning continents and long time periods. Such viruses may include divergent CCoVs identified in Sweden (88), Australia (89), and China (90–92). Notably, increased surveillance indicates CCoV-2c-like viruses may be the cause of ongoing winter waves of vomiting and diarrhea in dogs in the United Kingdom (93–95). Also of note is the finding that CCoVs with a highly recombinant spike gene derived from feline and canine coronavirus (96) have been isolated from humans and defined as human CCoV Z19 and CCoV-HuPn-2018, where they are considered to have respiratory tropism (1, 97–100). The recent full genome sequence of CCoV-UCD-1 [PP526172], first isolated in the 1970s and originally thought to have a TGEV-like NTD (101), is nearly identical to CCoV-HuPn-2018, adding further intrigue to these zoonotic-potential viruses.

CCoV-2a also exists as what are known as “pantropic” isolates. The initial isolate (CB/05) was responsible for a severe outbreak of fatal systemic disease in a pet shop in Bari, Italy, which included bronchopneumonia and neurological signs (102). These viruses have since been well reviewed in the literature (103) and have now been documented across the Mediterranean region over the past decades, as well as in other European countries (103). CB/05-like pantropic CCoVs are typified by severe clinical signs, lymphopenia, and infection of lymphoid tissue. While sequencing was limited at the time, we note that viruses clustering with CB/05 have also been historically detected in the United States (86). A recent evaluation of a localized outbreak from 2012 of severe enteritis in captive snow leopards used next-generation sequencing to identify a CB/05-like canine coronavirus present in the United States—further expanding the known distribution of these highly virulent viruses, as well as their capacity to infect felids without causing systemic disease (73). Data such as these raise the question of whether the recombination event that generates FCoV-2 occurs in felids or canids.

While mostly reported as clinical cases in domestic dogs, CCoV-2 is also widespread in wild canids (104) and non-canids (105), with a notable outbreak in red foxes in China between 2019 and 2022, caused by a virus from a distinct CCoV lineage (named Vu-CCoV) (106).

FCoV-23 is a recently emerged canine/feline recombinant virus that caused a large outbreak on the Mediterranean island of Cyprus during 2023, with (at the time of writing) documented spread of isolated travel-related cases in the United Kingdom (5, 7, 8). This is a concerning situation, as the virus is highly virulent, with most cats showing signs consistent with effusive FIP and a high degree of neurological signs, along with high viral loads in the colon—in cell types noted as having macrophage-like morphology. Compared to the other FCoV-2s that acquired a larger portion of their genome from CCoV-2, FCoV-23 only acquired its spike gene and a small region of Orf1b. The FCoV-23 spike gene has 97% identity to CCoV NA/09—a CB/05-like virus from Greece (107)—and is present in two forms, including one with a deletion of variable size (ranging from 456 to 735 bp, 152–245 amino acids, Fig. 1) in the NTD that results in a domain 0 truncated spike protein in the majority of studied cases. Notably, this “short” version of spike is highly fusogenic (108), but data on pathogenic outcomes are not yet available. As noted by Attipa et al., the reason behind this notable outbreak may be due to the “right mutation, right time, right place” theory (6), with major roles being played by both viral factors (such as recombination and the domain 0 deletion) and environmental/community factors (the large numbers of feral cats—up to 1.5 million—on a relatively small island).

CCoV-1 is typified by the isolate Elmö/02 (109), which has high identity to FCoV-1. This virus is not well understood, and—notably—likely cocirculates extensively with the various CCoV-2 viruses (110). This leads to challenges regarding surveillance efforts. To date, surveillance studies that include CCoV-1 have focused on the simultaneous amplification of a small region at the 5´ end of the S gene, which facilitates the distinction between CCoV-1, CCoV-2a, and CCoV-2b (110, 111). While these assays allow for the study of circulating genotypes and the detection of coinfections, variants with mutations or deletions in regions potentially related to pathogenicity and novel recombinants will be overlooked. The high percentage of coinfection between CCoV-1 and CCoV-2 reported in dogs in Europe (36.1%) (110) and Japan (41.5%) (112) suggests that the emergence of novel recombinants of CCoV is likely. Additionally, CCoV-1 contains a unique ORF3, upstream of the usual ORF3abc complex (113). The sequence of this ORF seems to be highly conserved (although only a handful of CCoV-1 variants have been studied), and its specific function remains unknown (113). The identification of atypical FCoVs with a truncated form of this ORF in cats (114) warrants further studies about the function of the protein encoded by this ORF and how often FCoV recombines with CCoV-1.

## TRANSMISSIBLE GASTROENTERITIS VIRUS (TGEV)

TGEV is an *Alphacoronavirus* closely related to CCoV that has historically caused widespread infections in pigs. It causes a fatal disease in neonatal piglets, due to selectively infecting and destroying enterocytes in the small intestine, causing diarrhea, vomiting, and dehydration, with mortality rates reaching up to 100% (115). In adults, it leads to transient fever, vomiting, and diarrhea, with much lower death rates. Additionally, sows infected with TGEV stop lactating (116, 117). Notably, TGEV variants also infect and replicate in the respiratory tract, but without causing overt respiratory disease (117), and are known as porcine respiratory coronavirus (PRCoV)—with these being a variant containing a deletion variable in size in the NTD (domain 0) of the spike protein (118, 119). This deletion has caused a remarkable change in cell tropism, causing TGEV to minimally infect enterocytes and replicate exclusively in the respiratory tract, without clinical signs (120). Interestingly, PRCoV infection induces antibodies that neutralize TGEV (121). This is because the deletion in the NTD of PRCoV occurs downstream of TGEV’s major inducer site of neutralizing antibodies (118, 120, 122). Therefore, TGEV infections tend to be milder when there is a coinfection with PRCoV, even though TGEV pathogenicity is not downregulated by the coinfection (119). TGEV/PRCoV strains cocirculate in swine herds, and recombinants with altered pathogenicity may occur (123).

### *Alphacoronavirus* domain 0 deletion/disruption hypothesis

While once considered unique to TGEV/PRCoV, the loss of the domain 0 through distinct and variable deletions now appears to be a more widespread phenomenon across the alphacoronaviruses. Notably, in the case of FCoV-23, domain 0 deletion appears to increase pathogenesis, not decrease it as expected for TGEV/PRCoV. It is also associated with certain clinical isolates of a phylogenetically distinct virus PEDV, which causes acute diarrhea, vomiting, and dehydration in pigs and is especially problematic in neonatal and suckling piglets, with high rates of mortality (124). In the late 2000s, new strains of PEDV arose in Asian countries, containing multiple insertions and deletions in the S gene, in comparison with the strains reported previously (125–127). Notably, indel PEDV strains can include deletions and insertions as small as 1 to 11 nucleotides. However, larger deletions have also been found in both cell culture-adapted and field strains of PEDV (termed S1 NTD-del PEDV), with sizes in line with those found for PRCoV and FCoV-23 short (194 to 216 amino acids) (128–133). Most publications report the existence of the S1 NTD-del PEDV alongside S-intact PEDV strains in farms where animals present acute diarrhea and vomiting, with no distinction between animals infected with either or both strains. However, Su and collaborators (134) report that animals infected with the S1 NTD-del PEDV strain shed less virus than those infected with the S-intact strain. Interestingly, coinfection between S-intact and S1 NTD-del PEDV strains appears to enhance the replication of the intact gene strain, due to mucin, bile, and bile acids positively acting on the replication of the S-intact strain, but not the S1 NTD-del strain. Additionally, although a 197 amino acid-deleted tissue culture-adapted strain (TC-PC177) infection in pigs resulted in milder clinical signs when compared to the highly pathogenic PC21A strain, prime inoculation with TC-PC177 did not confer immunity against PC21A, resulting in incomplete protection (135).

Domain 0 deletions ranging from 222 to 264 amino acids also occur for FCoV-1, specifically with the FIPV isolates C3663 (136), UU16 and UU21 (137), and UG-FH8/Karlslunde [ASU62499.1]. For FCoV-1, the generation and pathogenic outcome of truncated vs intact spikes currently remains unknown. In humans, the loss of domain 0 would appear to be related to the emergence of HCoV-229E from its bat ancestor, where the domain 0 is actually duplicated in some 229E-like bat viruses (138). For 229E-like viruses, the loss of domain 0 may be connected to a shift to respiratory tropism, as with PRCoV. Clearly, these large domain 0 deletions have variable and notable impact on virus pathogenesis that varies across species.

**Fig 2:**
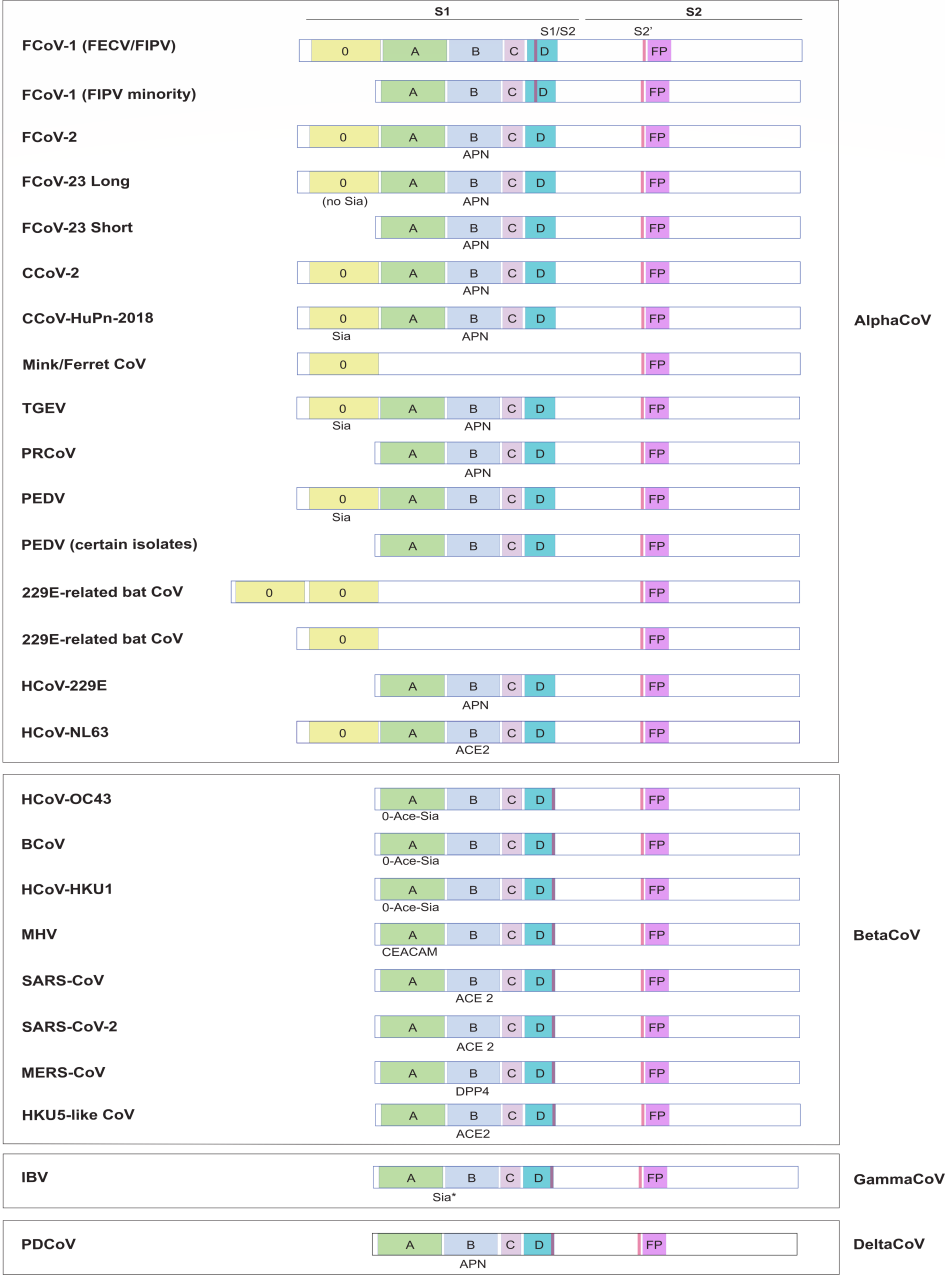
Graphical depiction of the spike proteins of selected alpha-, beta-, gamma-, and deltacoronaviruses. The location of the S1 and S2 subdomains is shown at the top. The 0, A, B, C, and D subdomains are in yellow, green, blue, light purple, and cyan, respectively. The fusion peptide (FP) is in magenta. The currently well-established receptors/attachment factors are shown below the region in spike with which they interact and are abbreviated as follows: Sia, sialic acid; O-Ace-Sia, O-acetylated sialic acid; ACE2, angiotensin-converting enzyme 2; CEACAM, carcinoembryonic antigen-related cell adhesion molecule. See reference 139.

Domain 0 has a lectin-like fold and appears to be a structural duplication of domain A (despite only 23% sequence identity) and often binds sialic acid. With TGEV, loss of pathogenicity was attributed to loss of sialic acid binding through loss of domain 0 (118, 119). However, FCoV-23 does not appear to bind sialic acid (108), implying a different mechanistic explanation linked more to the hyperfusogenic phenotype and rapid virus entry seen with the “short” spike in this case. Alphacoronaviruses and betacoronaviruses have a fundamentally different domain organization for their spikes (square-shaped vs V-shaped tertiary structure), and clearly, the presence or absence of the *Alphacoronavirus* spike domain 0 (which is missing in all betacoronaviruses) can have a major impact on viral tropism and pathogenesis, but currently without a predictable outcome. These deletions are summarized in Fig. 2.

## PERSPECTIVES

In this review, we address the genomic and pathogenic complexities within the alphacoronaviruses, with a focus on the species *Alphacoronavirus-1,* which consists of a group of significant pathogens of cats, dogs, and pigs. Recent findings have prompted a reanalysis of the overarching question of how we define virulence for these viruses, in particular for FCoV and CCoV. In addition to the distinct properties of FCoV-1 vs FCoV-2—with both able to convert to highly pathogenic forms—we need to address the question of whether “FIP/FIPV” just means robust macrophage tropism/spread, or whether it is a generalized systemic infection and a concomitant inflammatory response/cytokine storm. Notably, many FCoVs have extensive infection of lymphoid tissue that may not be picked up in traditional antibody-based immunohistochemistry approaches, for example, see reference 38. The detection of persistent or “sanctuary” sites that promote the long-term persistence of FCoV requires further investigation, as does more investigation of host genetic factors.

In the context of newly emerging viruses such as FCoV-23, we need to consider what exactly “FIP” is, clinically speaking; signs are already split into “wet” and “dry” manifestations, with dry FIP likely to be a much broader category than currently recognized. For FIP, are neurological manifestations (as with FCoV-23) just the tip of the iceberg? For example, rhinitis (44) and myocarditis (140, 141) have been documented, with infection possibly also leading to pancreatitis (although the latter is only well documented in ferrets infected with FRSCV; see reference 139). Other clinical conditions such as liver problems, stomatitis, etc. are also possible. Based on studies in pigs, animal alphacoronaviruses are often assumed to be enteric in nature. However, the deletion in the 0 domain of TGEV (PRCoV) and PEDV shows the impact viral mutations can have in cell tropism and viral pathogenicity. It also highlights the importance of viral monitoring and sequencing in livestock, as the emergence of highly pathogenic strains can cause not only substantial economic losses, but also a disruption in the food supply chain. While FCoV-2 and CCoV-2 are established enteric pathogens, recent findings of FCoV-1 in the respiratory tract of FIP cats (142), as well as in respiratory (143) and conjunctival (50) samples of cats without confirmed FIP, lead to a reconsideration of an enteric route of transmission for FCoV-1, despite the preponderance of viral RNA being shed in the feces. Without the ability to readily isolate viruses, it cannot be guaranteed that this viral RNA corresponds to infectious virions (144). The emergence of FCoV-23 has reinforced the need for more extensive surveillance and epidemiology of both FCoV and CCoV covering both genotypes, as well as the improved ability to study and manipulate FCoV-1 in cell culture, including identification of its receptor.

Recombination, along with mutations and generation of indels, is fundamental for coronavirus evolution—facilitating cross-species transmission and acting as a primary driver of viral spillover and emergence (145). Within the species *Alphacoronavirus-1,* there may exist a specific and dynamic “metavirome” (the sum of all viral genomes present in a sample) that is in a constant state of flux and can seed the emergence of both within-host and between-host variants with highly context-dependent properties. We propose that this selection of variants having discrete pathogenic properties is driven in fundamentally different ways between FCoV-1/CCoV-1 and FCoV-2/CCoV-2—by a process of accumulated point mutations/indels and recombination events, respectively. FCoV-CCoV may not be an individual entity, and we argue that using simple PCR methodologies for diagnosis and monitoring/surveillance may be treacherous—in that we are trying to hit a moving target. Thus, there is a need for robust sequencing that embraces the inherent sequence diversity and recombination that is part of the “lifestyle” of a coronavirus. We note that available commercial FIP-specific PCR-based tests have generally not been widely adopted in the marketplace as a successful tool for clinical diagnosis. Sequencing-based FIP tests are needed, especially in light of widespread antiviral drug use and the current lack of information on drug-resistant variants emerging.

As reported by LePoder (146), feline and canine coronaviruses have common genetic and pathobiological features, and it may be unwise to treat these viruses in an animal species-specific manner. This analogy also applies not only to the TGEV-like porcine viruses noted above, but also to coronaviruses of ferrets and mink—which also exist in different pathobiological forms, often with pyogranulomatous lesions and effusions remarkably similar to FIP in cats. These viruses are classified as either a separate subspecies (*Alphacoronavirus-2*) or subgenus (*Minacovirus*) and are notable pathogens of “exotics” in veterinary medicine (see reference 76)—but are poorly understood. Whether animals other than pigs—including wildlife species—harbor viruses that can readily recombine with FCoV/CCoV remains to be seen.

## References

[1] Keusch GT, Amuasi JH, Anderson DE, Daszak P, Eckerle I, Field H, Koopmans M, Lam SK, Das Neves CG, Peiris M, Perlman S, Wacharapluesadee S, Yadana S, Saif L. 2022. Pandemic origins and a One Health approach to preparedness and prevention: solutions based on SARS-CoV-2 and other RNA viruses. Proc Natl Acad Sci USA 119:e2202871119. doi:10.1073/pnas.2202871119 36215506 PMC9586299

[2] Jaimes JA, Millet JK, Stout AE, André NM, Whittaker GR. 2020. A tale of two viruses: the distinct spike glycoproteins of feline coronaviruses. Viruses 12:83. doi:10.3390/v12010083 31936749 PMC7019228

[3] Belouzard S, Millet JK, Licitra BN, Whittaker GR. 2012. Mechanisms of coronavirus cell entry mediated by the viral spike protein. Viruses 4:1011–1033. doi:10.3390/v4061011 22816037 PMC3397359

[4] Gao Y-Y, Wang Q, Liang X-Y, Zhang S, Bao D, Zhao H, Li S-B, Wang K, Hu G-X, Gao F-S. 2023. An updated review of feline coronavirus: mind the two biotypes. Virus Res 326:199059. doi:10.1016/j.virusres.2023.199059 36731629 PMC10194308

[5] Attipa C, Gunn-Moore D, Mazeri S, Epaminondas D, Lyraki M, Hardas A, Loukaidou S, Gentil M. 2023. Concerning feline infectious peritonitis outbreak in Cyprus. Vet Rec 192:449–450. doi:10.1002/vetr.3143 37265279

[6] Attipa C, Warr AS, Epaminondas D, O’Shea M, Hanton AJ, Fletcher S, Malbon A, Lyraki M, Hammond R, Hardas A, Zanti A, Loukaidou S, Gentil M, Gunn-Moore D, Lycett SJ, Mazeri S, Tait-Burkard C. 2025. Feline infectious peritonitis epizootic caused by a recombinant coronavirus. Nature. doi:10.1038/s41586-025-09340-0 PMC12408369 40633571

[7] Hardas A, Attipa C, Gunn-Moore D, Epaminondas D, Lyraki M, Gentil M, Loukaidou S, Mazeri S. 2023. Widespread outbreak of FIP in Cyprus with a suspected highly virulent feline coronavirus strain. Vet Times 53:23.

[8] Warr A, Attipa C, Gunn-Moore D, Tait-Burkard C. 2023. FCoV-23 causing FIP in a cat imported to the UK from Cyprus. Vet Rec 193:414–415. doi:10.1002/vetr.3696 37975439

[9] Ettinger SJ, Co︢té E, Feldman EC. 2024. Textbook of veterinary internal medicine: diseases of the dog and cat. 9th ed. Elsevier, St. Louis, MO.

[10] Pedersen NC, Sykes JE. 2021. Chapter 31, Feline coronavirus infections, p 360–381. In Sykes JE (ed), Greene’s infectious diseases of the dog and cat, 5th ed. Elsevier, London.

[11] Pedersen NC. 1995. An overview of feline enteric coronavirus and infectious peritonitis virus infections. Feline Pract.

[12] AVMA 2022. AVMA pet ownership and demographics sourcebook. American Veterinary Medical Association, Schaumburg, IL.

[13] Thayer V, Gogolski S, Felten S, Hartmann K, Kennedy M, Olah GA. 2022. 2022 AAFP/EveryCat feline infectious peritonitis diagnosis guidelines. J Feline Med Surg 24:905–933. doi:10.1177/1098612X221118761 36002137 PMC10812230

[14] Vennema H, Poland A, Foley J, Pedersen NC. 1998. Feline infectious peritonitis viruses arise by mutation from endemic feline enteric coronaviruses. Virology (Auckland) 243:150–157. doi:10.1006/viro.1998.9045 PMC7131759 9527924

[15] Tasker S, Addie DD, Egberink H, Hofmann-Lehmann R, Hosie MJ, Truyen U, Belák S, Boucraut-Baralon C, Frymus T, Lloret A, Marsilio F, Pennisi MG, Thiry E, Möstl K, Hartmann K. 2023. Feline infectious peritonitis: European advisory board on cat diseases guidelines. Viruses 15:1847. doi:10.3390/v15091847 37766254 PMC10535984

[16] Barker EN, Stranieri A, Helps CR, Porter EL, Davidson AD, Day MJ, Knowles T, Kipar A, Tasker S. 2017. Limitations of using feline coronavirus spike protein gene mutations to diagnose feline infectious peritonitis. Vet Res 48:60. doi:10.1186/s13567-017-0467-9 28982390 PMC5629788

[17] Jähne S, Felten S, Bergmann M, Erber K, Matiasek K, Meli ML, Hofmann-Lehmann R, Hartmann K. 2022. Detection of feline coronavirus variants in cats without feline infectious peritonitis. Viruses 14:1671. doi:10.3390/v14081671 36016293 PMC9412601

[18] Licitra BN, Millet JK, Regan AD, Hamilton BS, Rinaldi VD, Duhamel GE, Whittaker GR. 2013. Mutation in spike protein cleavage site and pathogenesis of feline coronavirus. Emerg Infect Dis 19:1066–1073. doi:10.3201/eid1907.121094 23763835 PMC3713968

[19] Licitra BN, Sams KL, Lee DW, Whittaker GR. 2014. Feline coronaviruses associated with feline infectious peritonitis have modifications to spike protein activation sites at two discrete positions. arXiv. doi:10.48550/arXiv.1412.4034

[20] Barker EN, Tasker S. 2020. Advances in molecular diagnostics and treatment of feline infectious peritonitis. Adv Small Anim Care 1:161–188. doi:10.1016/j.yasa.2020.07.011

[21] Zehr JD, Kosakovsky Pond SL, Millet JK, Olarte-Castillo XA, Lucaci AG, Shank SD, Ceres KM, Choi A, Whittaker GR, Goodman LB, Stanhope MJ. 2023. Natural selection differences detected in key protein domains between non-pathogenic and pathogenic feline coronavirus phenotypes. Virus Evol 9:vead019. doi:10.1093/ve/vead019 37038392 PMC10082545

[22] Barker EN, Tasker S, Gruffydd-Jones TJ, Tuplin CK, Burton K, Porter E, Day MJ, Harley R, Fews D, Helps CR, Siddell SG. 2013. Phylogenetic analysis of feline coronavirus strains in an epizootic outbreak of feline infectious peritonitis. J Vet Intern Med 27:445–450. doi:10.1111/jvim.12058 23517431 PMC7166722

[23] Healey EA, Andre NM, Miller AD, Whittaker GR, Berliner EA. 2022. Outbreak of feline infectious peritonitis (FIP) in shelter-housed cats: molecular analysis of the feline coronavirus S1/S2 cleavage site consistent with a ‘circulating virulent–avirulent theory’ of FIP pathogenesis. J Feline Med Surg Open Rep 8:20551169221074226. doi:10.1177/20551169221074226 PMC8841931 35173971

[24] Wang Y-T, Su B-L, Hsieh L-E, Chueh L-L. 2013. An outbreak of feline infectious peritonitis in a Taiwanese shelter: epidemiologic and molecular evidence for horizontal transmission of a novel type II feline coronavirus. Vet Res 44:57. doi:10.1186/1297-9716-44-57 23865689 PMC3720556

[25] Hickman MA, Morris JG, Rogers QR, Pedersen NC. 1995. Elimination of feline coronavirus infection from a large experimental specific pathogen-free cat breeding colony by serologic testing and isolation. Feline Pract 23:96–102.

[26] Potkay S, Bacher JD, Pitts TW. 1974. Feline infectious peritonitis in a closed breeding colony. Lab Anim Sci 24:279–289. 4362875

[27] Vennema H, de Groot RJ, Harbour DA, Dalderup M, Gruffydd-Jones T, Horzinek MC, Spaan WJ. 1990. Early death after feline infectious peritonitis virus challenge due to recombinant vaccinia virus immunization. J Virol 64:1407–1409. doi:10.1128/JVI.64.3.1407-1409.1990 2154621 PMC249267

[28] Hohdatsu T, Nakamura M, Ishizuka Y, Yamada H, Koyama H. 1991. A study on the mechanism of antibody-dependent enhancement of feline infectious peritonitis virus infection in feline macrophages by monoclonal antibodies. Arch Virol 120:207–217. doi:10.1007/BF01310476 1659798 PMC7087175

[29] Corapi WV, Olsen CW, Scott FW. 1992. Monoclonal antibody analysis of neutralization and antibody-dependent enhancement of feline infectious peritonitis virus. J Virol 66:6695–6705. doi:10.1128/JVI.66.11.6695-6705.1992 1383568 PMC240165

[30] Weiss RC, Scott FW. 1981. Antibody-mediated enhancement of disease in feline infectious peritonitis: comparisons with dengue hemorrhagic fever. Comp Immunol Microbiol Infect Dis 4:175–189. doi:10.1016/0147-9571(81)90003-5 6754243 PMC7134169

[31] Whittaker GR, André NM, Millet JK. 2018. Improving virus taxonomy by recontextualizing sequence-based classification with biologically relevant data: the case of the Alphacoronavirus 1 species. mSphere 3:e00463-17. doi:10.1128/mSphereDirect.00463-17 29299531 PMC5750389

[32] Choi A, Kots ED, Singleton DT, Weinstein H, Whittaker GR. 2024. Analysis of the molecular determinants for furin cleavage of the spike protein S1/S2 site in defined strains of the prototype coronavirus murine hepatitis virus (MHV). Virus Res 340:199283. doi:10.1016/j.virusres.2023.199283 38043726 PMC10755501

[33] Le Coupanec A, Desforges M, Meessen-Pinard M, Dubé M, Day R, Seidah NG, Talbot PJ. 2015. Cleavage of a neuroinvasive human respiratory virus spike glycoprotein by proprotein convertases modulates neurovirulence and virus spread within the central nervous system. PLoS Pathog 11:e1005261. doi:10.1371/journal.ppat.1005261 26545254 PMC4636366

[34] Lamers MM, Mykytyn AZ, Breugem TI, Wang Y, Wu DC, Riesebosch S, van den Doel PB, Schipper D, Bestebroer T, Wu NC, Haagmans BL. 2021. Human airway cells prevent SARS-CoV-2 multibasic cleavage site cell culture adaptation. Elife 10:e66815. doi:10.7554/eLife.66815 33835028 PMC8131099

[35] Sasaki M, Uemura K, Sato A, Toba S, Sanaki T, Maenaka K, Hall WW, Orba Y, Sawa H. 2021. SARS-CoV-2 variants with mutations at the S1/S2 cleavage site are generated in vitro during propagation in TMPRSS2-deficient cells. PLoS Pathog 17:e1009233. doi:10.1371/journal.ppat.1009233 33476327 PMC7853460

[36] Pedersen NC, Evermann JF, McKeirnan AJ, Ott RL. 1984. Pathogenicity studies of feline coronavirus isolates 79-1146 and 79-1683. Am J Vet Res 45:2580–2585. doi:10.2460/ajvr.1984.45.12.2580 6084432

[37] Takano T, Kawakami C, Yamada S, Satoh R, Hohdatsu T. 2008. Antibody-dependent enhancement occurs upon re-infection with the identical serotype virus in feline infectious peritonitis virus infection. J Vet Med Sci 70:1315–1321. doi:10.1292/jvms.70.1315 19122397

[38] Sweet A, Andre N, Licitra BN, Whittaker G. 2022. RNA in-situ hybridization for pathology-based diagnosis of feline infectious peritonitis (FIP): current diagnostics for FIP and comparison to the current gold standard. Qeios 4. doi:10.32388/NUN8KB.2

[39] Kipar A, Meli ML, Baptiste KE, Bowker LJ, Lutz H. 2010. Sites of feline coronavirus persistence in healthy cats. J Gen Virol 91:1698–1707. doi:10.1099/vir.0.020214-0 20237226

[40] Ehmann R, Kristen-Burmann C, Bank-Wolf B, König M, Herden C, Hain T, Thiel H-J, Ziebuhr J, Tekes G. 2018. Reverse genetics for type I feline coronavirus field isolate to study the molecular pathogenesis of feline infectious peritonitis. mBio 9:e01422-18. doi:10.1128/mBio.01422-18 30065095 PMC6069117

[41] Ouyang H, Liu J, Yin Y, Cao S, Yan R, Ren Y, Zhou D, Li Q, Li J, Liao X, Ji W, Du B, Si Y, Hu C. 2022. Epidemiology and comparative analyses of the s gene on feline coronavirus in central China. Pathogens 11:460. doi:10.3390/pathogens11040460 35456135 PMC9031646

[42] Murphy BG, Castillo D, Neely NE, Kol A, Brostoff T, Grant CK, Reagan KL. 2024. Serologic, virologic and pathologic features of cats with naturally occurring feline infectious peritonitis enrolled in antiviral clinical trials. Viruses 16:462. doi:10.3390/v16030462 38543827 PMC10975727

[43] André NM, Cossic B, Davies E, Miller AD, Whittaker GR. 2019. Distinct mutation in the feline coronavirus spike protein cleavage activation site in a cat with feline infectious peritonitis-associated meningoencephalomyelitis. J Feline Med Surg Open Rep 5:2055116919856103. doi:10.1177/2055116919856103 PMC6739741 31534775

[44] André NM, Miller AD, Whittaker GR. 2020. Feline infectious peritonitis virus-associated rhinitis in a cat. JFMS Open Rep 6:2055116920930582. doi:10.1177/2055116920930582 32637147 PMC7313338

[45] Thomas G. 2002. Furin at the cutting edge: from protein traffic to embryogenesis and disease. Nat Rev Mol Cell Biol 3:753–766. doi:10.1038/nrm934 12360192 PMC1964754

[46] Tian S, Huajun W, Wu J. 2012. Computational prediction of furin cleavage sites by a hybrid method and understanding mechanism underlying diseases. Sci Rep 2:261. doi:10.1038/srep00261 22355773 PMC3281273

[47] Turk B. 2006. Targeting proteases: successes, failures and future prospects. Nat Rev Drug Discov 5:785–799. doi:10.1038/nrd2092 16955069

[48] Choi A, Frazier LE, Olarte-Castillo XA, Whittaker G. 2024. The furin cleavage site of feline coronavirus type 1 (FCoV-1) and its structural localization within the S1 domain D. Qeios. doi:10.32388/LY79Y2.2

[49] Millet JK, Whittaker GR. 2015. Host cell proteases: critical determinants of coronavirus tropism and pathogenesis. Virus Res 202:120–134. doi:10.1016/j.virusres.2014.11.021 25445340 PMC4465284

[50] Olarte-Castillo XA, Licitra BN, André NM, Sierra MA, Mason CE, Goodman LB, Whittaker GR. 2023. Intra-host variation in the spike S1/S2 region of a feline coronavirus type-1 in a cat with persistent infection. bioRxiv:2023.07.31.551356. doi:10.1101/2023.07.31.551356

[51] Vignuzzi M, Stone JK, Arnold JJ, Cameron CE, Andino R. 2006. Quasispecies diversity determines pathogenesis through cooperative interactions in a viral population. Nature 439:344–348. doi:10.1038/nature04388 16327776 PMC1569948

[52] Fraser C, Lythgoe K, Leventhal GE, Shirreff G, Hollingsworth TD, Alizon S, Bonhoeffer S. 2014. Virulence and pathogenesis of HIV-1 infection: an evolutionary perspective. Science 343:1243727. doi:10.1126/science.1243727 24653038 PMC5034889

[53] Raghwani J, Wu C-H, Ho CKY, De Jong M, Molenkamp R, Schinkel J, Pybus OG, Lythgoe KA. 2019. High-resolution evolutionary analysis of within-host hepatitis C virus infection. J Infect Dis 219:1722–1729. doi:10.1093/infdis/jiy747 30602023 PMC6500553

[54] Lythgoe KA, Hall M, Ferretti L, de Cesare M, MacIntyre-Cockett G, Trebes A, Andersson M, Otecko N, Wise EL, Moore N, et al.. 2021. SARS-CoV-2 within-host diversity and transmission. Science 372:eabg0821. doi:10.1126/science.abg0821 33688063 PMC8128293

[55] Hoetelmans RM. 1998. Sanctuary sites in HIV-1 infection. Antivir Ther 3:13–17. 10723504

[56] Bons E, Regoes RR. 2018. Virus dynamics and phyloanatomy: merging population dynamic and phylogenetic approaches. Immunol Rev 285:134–146. doi:10.1111/imr.12688 30129202

[57] Lorenzo-Redondo R, Fryer HR, Bedford T, Kim E-Y, Archer J, Pond SLK, Chung Y-S, Penugonda S, Chipman J, Fletcher CV, Schacker TW, Malim MH, Rambaut A, Haase AT, McLean AR, Wolinsky SM. 2016. Persistent HIV-1 replication maintains the tissue reservoir during therapy. Nature 530:51–56. doi:10.1038/nature16933 26814962 PMC4865637

[58] Normandin E, Rudy M, Barkas N, Schaffner SF, Levine Z, Padera RF, Babadi M, Mukerji SS, Park DJ, MacInnis BL, Siddle KJ, Sabeti PC, Solomon IH. 2023. High-depth sequencing characterization of viral dynamics across tissues in fatal COVID-19 reveals compartmentalized infection. Nat Commun 14:574. doi:10.1038/s41467-022-34256-y 36732505 PMC9894515

[59] Zhang S. 2020. A much-hyped COVID-19 drug is almost identical to a black-market cat cure. The Atlantic. Available from: https://www.theatlantic.com/science/archive/2020/05/remdesivir-cats/611341. Retrieved 02 Jul 2024.

[60] Sase O. 2023. Molnupiravir treatment of 18 cats with feline infectious peritonitis: a case series. J Vet Intern Med 37:1876–1880. doi:10.1111/jvim.16832 37551843 PMC10472991

[61] Zuzzi-Krebitz A-M, Buchta K, Bergmann M, Krentz D, Zwicklbauer K, Dorsch R, Wess G, Fischer A, Matiasek K, Hönl A, Fiedler S, Kolberg L, Hofmann-Lehmann R, Meli ML, Spiri AM, Helfer-Hungerbuehler AK, Felten S, Zablotski Y, Alberer M, Both U von, Hartmann K. 2024. Short treatment of 42 days with oral GS-441524 results in equal efficacy as the recommended 84-day treatment in cats suffering from feline infectious peritonitis with effusion—a prospective randomized controlled study. Viruses 16:1144. doi:10.3390/v16071144 39066306 PMC11281457

[62] Coggins SJ, Norris JM, Malik R, Govendir M, Hall EJ, Kimble B, Thompson MF. 2023. Outcomes of treatment of cats with feline infectious peritonitis using parenterally administered remdesivir, with or without transition to orally administered GS-441524. J Vet Intern Med 37:1772–1783. doi:10.1111/jvim.16803 37439383 PMC10473006

[63] Kosakovsky Pond SL, Martin D. 2023. Anti-COVID drug accelerates viral evolution. Nature 623:486–487. doi:10.1038/d41586-023-03248-3 37875683

[64] Sanderson T, Hisner R, Donovan-Banfield I, Hartman H, Løchen A, Peacock TP, Ruis C. 2023. A molnupiravir-associated mutational signature in global SARS-CoV-2 genomes. Nature 623:594–600. doi:10.1038/s41586-023-06649-6 37748513 PMC10651478

[65] Chomont N. 2023. Silence, escape and survival drive the persistence of HIV. Nature 614:236–237. doi:10.1038/d41586-022-04492-9 36599993

[66] Irwin KK, Renzette N, Kowalik TF, Jensen JD. 2016. Antiviral drug resistance as an adaptive process. Virus Evol 2:vew014. doi:10.1093/ve/vew014 28694997 PMC5499642

[67] Tusell SM, Schittone SA, Holmes KV. 2007. Mutational analysis of aminopeptidase N, a receptor for several group 1 coronaviruses, identifies key determinants of viral host range. J Virol 81:1261–1273. doi:10.1128/JVI.01510-06 17093189 PMC1797531

[68] Lin C-N, Su B-L, Wang C-H, Hsieh M-W, Chueh T-J, Chueh L-L. 2009. Genetic diversity and correlation with feline infectious peritonitis of feline coronavirus type I and II: a 5-year study in Taiwan. Vet Microbiol 136:233–239. doi:10.1016/j.vetmic.2008.11.010 19117699 PMC7117496

[69] Tresnan DB, Levis R, Holmes KV. 1996. Feline aminopeptidase N serves as a receptor for feline, canine, porcine, and human coronaviruses in serogroup I. J Virol 70:8669–8674. doi:10.1128/JVI.70.12.8669-8674.1996 8970993 PMC190961

[70] An D-J, Jeoung H-Y, Jeong W, Park J-Y, Lee M-H, Park B-K. 2011. Prevalence of Korean cats with natural feline coronavirus infections. Virol J 8:455. doi:10.1186/1743-422X-8-455 21951835 PMC3219666

[71] Shiba N, Maeda K, Kato H, Mochizuki M, Iwata H. 2007. Differentiation of feline coronavirus type I and II infections by virus neutralization test. Vet Microbiol 124:348–352. doi:10.1016/j.vetmic.2007.04.031 17543480 PMC7117252

[72] McArdle F, Bennett M, Gaskell RM, Tennant B, Kelly DF, Gaskell CJ. 1992. Induction and enhancement of feline infectious peritonitis by canine coronavirus. ajvr 53:1500–1506. doi:10.2460/ajvr.1992.53.09.1500 1329586

[73] Olarte-Castillo XA, Schlecht AB, Calle PP, Whittaker GR. 2025. An outbreak of canine coronavirus type 2 in captive snow leopards (Panthera uncia) demonstrates a possible role for felids as mixing vessels for alphacoronaviruses. IJID One Health 7:100057. doi:10.1016/j.ijidoh.2025.100057

[74] Regan AD, Shraybman R, Cohen RD, Whittaker GR. 2008. Differential role for low pH and cathepsin-mediated cleavage of the viral spike protein during entry of serotype II feline coronaviruses. Vet Microbiol 132:235–248. doi:10.1016/j.vetmic.2008.05.019 18606506 PMC2588466

[75] Wicht O, Li W, Willems L, Meuleman TJ, Wubbolts RW, van Kuppeveld FJM, Rottier PJM, Bosch BJ. 2014. Proteolytic activation of the porcine epidemic diarrhea coronavirus spike fusion protein by trypsin in cell culture. J Virol 88:7952–7961. doi:10.1128/JVI.00297-14 24807723 PMC4097775

[76] Tarbert DK, Bolin LL, Stout AE, Schaefer DMW, Ruby RE, Rodriguez-Ramos Fernandez J, Duhamel GE, Whittaker GR, de Matos R. 2020. Persistent infection and pancytopenia associated with ferret systemic coronaviral disease in a domestic ferret. J Vet Diagn Invest 32:616–620. doi:10.1177/1040638720937105 32589111 PMC7438646

[77] Wang N, Ji W, Jiao H, Veit M, Sun J, Wang Y, Ma X, Wang Y, Wang Y, Li X-X, Zhang X, Chen J, Wei J, Xu Y, Guo D, Zhai X, Merits A, Li C, Rey FA, Dobrikov GM, Gao GF, Zhang S, Bi Y, Su S. 2025. A MERS-CoV-like mink coronavirus uses ACE2 as an entry receptor. Nature 642:739–746. doi:10.1038/s41586-025-09007-w 40306315

[78] Stout AE, Guo Q, Millet JK, de Matos R, Whittaker GR. 2021. Coronaviruses associated with the superfamily Musteloidea. mBio 12:e02873-20. doi:10.1128/mBio.02873-20 33468694 PMC7845646

[79] Larsen AE, Gorham JR. 1975. A new mink enteritis: an initial report, p 291–292. In Vm/sac, veterinary medicine & small animal clinician. Vol. 70. 1038885

[80] Martínez J, Reinacher M, Perpiñán D, Ramis A. 2008. Identification of group 1 coronavirus antigen in multisystemic granulomatous lesions in ferrets (Mustela putorius furo). J Comp Pathol 138:54–58. doi:10.1016/j.jcpa.2007.10.002 18067916 PMC7094249

[81] Williams BH, Kiupel M, West KH, Raymond JT, Grant CK, Glickman LT. 2000. Coronavirus-associated epizootic catarrhal enteritis in ferrets. J Am Vet Med Assoc 217:526–530. doi:10.2460/javma.2000.217.526 10953717

[82] Minami S, Kuroda Y, Terada Y, Yonemitsu K, Van Nguyen D, Kuwata R, Shimoda H, Takano A, Maeda K. 2016. Detection of novel ferret coronaviruses and evidence of recombination among ferret coronaviruses. Virus Genes 52:858–862. doi:10.1007/s11262-016-1365-3 27369429 PMC7088552

[83] Lamers MM, Smits SL, Hundie GB, Provacia LB, Koopmans M, Osterhaus ADME, Haagmans BL, Raj VS. 2016. Naturally occurring recombination in ferret coronaviruses revealed by complete genome characterization. J Gen Virol 97:2180–2186. doi:10.1099/jgv.0.000520 27283016 PMC7079585

[84] Erles K, Brownlie J. 2008. Canine respiratory coronavirus: an emerging pathogen in the canine infectious respiratory disease complex. Vet Clin North Am Small Anim Pract 38:815–825. doi:10.1016/j.cvsm.2008.02.008 18501280 PMC7114852

[85] Priestnall SL, Mitchell JA, Walker CA, Erles K, Brownlie J. 2014. New and emerging pathogens in canine infectious respiratory disease. Vet Pathol 51:492–504. doi:10.1177/0300985813511130 24232191

[86] Licitra BN, Whittaker GR, Dubovi EJ, Duhamel GE. 2014. Genotypic characterization of canine coronaviruses associated with fatal canine neonatal enteritis in the United States. J Clin Microbiol 52:4230–4238. doi:10.1128/JCM.02158-14 25253797 PMC4313292

[87] Regan AD, Millet JK, Tse LPV, Chillag Z, Rinaldi VD, Licitra BN, Dubovi EJ, Town CD, Whittaker GR. 2012. Characterization of a recombinant canine coronavirus with a distinct receptor-binding (S1) domain. Virology (Auckland) 430:90–99. doi:10.1016/j.virol.2012.04.013 PMC3377836 22609354

[88] Escutenaire S, Isaksson M, Renström LHM, Klingeborn B, Buonavoglia C, Berg M, Belák S, Thorén P. 2007. Characterization of divergent and atypical canine coronaviruses from Sweden. Arch Virol 152:1507–1514. doi:10.1007/s00705-007-0986-1 17533554 PMC7087124

[89] Naylor MJ, Walia CS, McOrist S, Lehrbach PR, Deane EM, Harrison GA. 2002. Molecular characterization confirms the presence of a divergent strain of canine coronavirus (UWSMN-1) in Australia. J Clin Microbiol 40:3518–3522. doi:10.1128/JCM.40.9.3518-3522.2002 12202609 PMC130832

[90] Chen S, Liu D, Tian J, Kang H, Guo D, Jiang Q, Liu J, Li Z, Hu X, Qu L. 2019. Molecular characterization of HLJ-073, a recombinant canine coronavirus strain from China with an ORF3abc deletion. Arch Virol 164:2159–2164. doi:10.1007/s00705-019-04296-9 31152250 PMC7086736

[91] He H-J, Zhang W, Liang J, Lu M, Wang R, Li G, He J-W, Chen J, Chen J, Xing G, Chen Y. 2020. Etiology and genetic evolution of canine coronavirus circulating in five provinces of China, during 2018–2019. Microb Pathog 145:104209. doi:10.1016/j.micpath.2020.104209 32311431 PMC7165111

[92] Wu S, He X, Zhang B, An L, You L, Luo S, Yang F, Pei X, Chen J. 2023. Molecular epidemiology and genetic diversity of canine coronavirus from domestic dogs in Chengdu, China from 2020 to 2021 using a multiplex RT-PCR. Infect Genet Evol 112:105463. doi:10.1016/j.meegid.2023.105463 37295484

[93] Radford AD, Singleton DA, Jewell C, Appleton C, Rowlingson B, Hale AC, Cuartero CT, Newton R, Sánchez-Vizcaíno F, Greenberg D, Brant B, Bentley EG, Stewart JP, Smith S, Haldenby S, Noble P-JM, Pinchbeck GL. 2021. Outbreak of severe vomiting in dogs associated with a canine enteric coronavirus, United Kingdom. Emerg Infect Dis 27:517–528. doi:10.3201/eid2702.202452 33496240 PMC7853541

[94] Stavisky J, Pinchbeck G, Gaskell RM, Dawson S, German AJ, Radford AD. 2012. Cross sectional and longitudinal surveys of canine enteric coronavirus infection in kennelled dogs: a molecular marker for biosecurity. Infect Genet Evol 12:1419–1426. doi:10.1016/j.meegid.2012.04.010 22543007 PMC7106024

[95] Cunningham-Oakes E, Pilgrim J, Darby AC, Appleton C, Jewell C, Rowlingson B, Cuartero CT, Newton R, Sánchez-Vizcaíno F, Fins IS, Brant B, Smith S, Penrice-Randal R, Clegg SR, Roberts APE, Millson SH, Pinchbeck GL, Noble P-JM, Radford AD. 2024. Emerging variants of canine enteric coronavirus associated with outbreaks of gastroenteric disease. Emerg Infect Dis 30:1240–1244. doi:10.3201/eid3006.231184 38782018 PMC11139001

[96] Zehr JD, Pond SLK, Martin DP, Ceres K, Whittaker GR, Millet JK, Goodman LB, Stanhope MJ. 2022. Recent zoonotic spillover and tropism shift of a canine coronavirus is associated with relaxed selection and putative loss of function in NTD subdomain of spike protein. Viruses 14:853. doi:10.3390/v14050853 35632597 PMC9145938

[97] Buonavoglia A, Pellegrini F, Decaro N, Galgano M, Pratelli A. 2023. A one health perspective on canine coronavirus: a wolf in sheep’s clothing? Microorganisms 11:921. doi:10.3390/microorganisms11040921 37110344 PMC10143937

[98] Pratelli A, Buonavoglia A, Lanave G, Tempesta M, Camero M, Martella V, Decaro N. 2021. One world, one health, one virology of the mysterious labyrinth of coronaviruses: the canine coronavirus affair. Lancet Microbe 2:e646–e647. doi:10.1016/S2666-5247(21)00282-2 34778852 PMC8577845

[99] Lednicky JA, Tagliamonte MS, White SK, Blohm GM, Alam MM, Iovine NM, Salemi M, Mavian C, Morris JG. 2022. Isolation of a novel recombinant canine coronavirus from a visitor to haiti: further evidence of transmission of coronaviruses of zoonotic origin to humans. Clin Infect Dis 75:e1184–e1187. doi:10.1093/cid/ciab924 34718467 PMC9402678

[100] Vlasova AN, Diaz A, Damtie D, Xiu L, Toh T-H, Lee JS-Y, Saif LJ, Gray GC. 2022. Novel canine coronavirus isolated from a hospitalized patient with pneumonia in east Malaysia. Clin Infect Dis 74:446–454. doi:10.1093/cid/ciab456 34013321 PMC8194511

[101] Wesley RD. 1999. The S gene of canine coronavirus, strain UCD-1, is more closely related to the S gene of transmissible gastroenteritis virus than to that of feline infectious peritonitis virus. Virus Res 61:145–152. doi:10.1016/s0168-1702(99)00032-5 10475084 PMC7126756

[102] Buonavoglia C, Decaro N, Martella V, Elia G, Campolo M, Desario C, Castagnaro M, Tempesta M. 2006. Canine coronavirus highly pathogenic for dogs. Emerg Infect Dis 12:492–494. doi:10.3201/eid1203.050839 16704791 PMC3291441

[103] Decaro N, Buonavoglia C. 2011. Canine coronavirus: not only an enteric pathogen. Vet Clin North Am Small Anim Pract 41:1121–1132. doi:10.1016/j.cvsm.2011.07.005 22041207 PMC7114679

[104] Whittaker G, Stout A. 2022. Coronaviruses in wild canids: a review of the literature. Qeios. doi:10.32388/5UZPYI

[105] Olarte-Castillo XA, Dos Remédios JF, Heeger F, Hofer H, Karl S, Greenwood AD, East ML. 2021. The virus–host interface: molecular interactions of Alphacoronavirus-1 variants from wild and domestic hosts with mammalian aminopeptidase N. Mol Ecol 30:2607–2625. doi:10.1111/mec.15910 33786949 PMC8251223

[106] Liu Y, Deng Y, Niu S, Zhu N, Song J, Zhang X, Su W, Nie W, Lu R, Irwin DM, Gao GF, Wang W, Wang Q, Tan W, Zhang S. 2023. Discovery and identification of a novel canine coronavirus causing a diarrhea outbreak in Vulpes. Sci Bull Sci Found Philipp 68:2598–2606. doi:10.1016/j.scib.2023.09.011 37758615

[107] Ntafis V, Xylouri E, Mari V, Papanastassopoulou M, Papaioannou N, Thomas A, Buonavoglia C, Decaro N. 2012. Molecular characterization of a canine coronavirus NA/09 strain detected in a dog’s organs. Arch Virol 157:171–175. doi:10.1007/s00705-011-1141-6 22002680 PMC7087105

[108] Tortorici MA, Choi A, Gibson CA, Lee J, Brown JT, Stewart C, Joshi A, Harari S, Willoughby I, Treichel C, Leaf EM, Bloom JD, King NP, Tait-Burkard C, Whittaker GR, Veesler D. 2025. Loss of FCoV-23 spike domain 0 enhances fusogenicity and entry kinetics. Nature. doi:10.1038/s41586-025-09155-z PMC12408340 40634609

[109] Pratelli A, Martella V, Decaro N, Tinelli A, Camero M, Cirone F, Elia G, Cavalli A, Corrente M, Greco G, Buonavoglia D, Gentile M, Tempesta M, Buonavoglia C. 2003. Genetic diversity of a canine coronavirus detected in pups with diarrhoea in Italy. J Virol Methods 110:9–17. doi:10.1016/s0166-0934(03)00081-8 12757915 PMC7119961

[110] Decaro N, Mari V, Elia G, Addie DD, Camero M, Lucente MS, Martella V, Buonavoglia C. 2010. Recombinant canine coronaviruses in dogs, Europe. Emerg Infect Dis 16:41–47. doi:10.3201/eid1601.090726 20031041 PMC2874359

[111] Erles K, Brownlie J. 2009. Sequence analysis of divergent canine coronavirus strains present in a UK dog population. Virus Res 141:21–25. doi:10.1016/j.virusres.2008.12.009 19162099 PMC7114384

[112] Soma T, Ohinata T, Ishii H, Takahashi T, Taharaguchi S, Hara M. 2011. Detection and genotyping of canine coronavirus RNA in diarrheic dogs in Japan. Res Vet Sci 90:205–207. doi:10.1016/j.rvsc.2010.05.027 20557915 PMC7118793

[113] Lorusso A, Decaro N, Schellen P, Rottier PJM, Buonavoglia C, Haijema B-J, de Groot RJ. 2008. Gain, preservation, and loss of a group 1a coronavirus accessory glycoprotein. J Virol 82:10312–10317. doi:10.1128/JVI.01031-08 18667517 PMC2566247

[114] Le Poder S, Pham-Hung d’Alexandry d’Orangiani A-L, Duarte L, Fournier A, Horhogea C, Pinhas C, Vabret A, Eloit M. 2013. Infection of cats with atypical feline coronaviruses harbouring a truncated form of the canine type I non-structural ORF3 gene. Infect Genet Evol 20:488–494. doi:10.1016/j.meegid.2013.09.024 24121017 PMC7106123

[115] Garwes DJ. 1988. Transmissible gastroenteritis. Vet Rec 122:462–463. doi:10.1136/vr.122.19.462 2839932

[116] Saif LJ. 1999. Comparative pathogenesis of enteric viral infections of swine, p 47–59. In Paul PS, Francis DH (ed), Mechanisms in the pathogenesis of enteric diseases 2. Springer US, Boston, MA. 10.1007/978-1-4615-4143-1_4 10659343

[117] Kemeny LJ, Wiltsey VL, Riley JL. 1975. Upper respiratory infection of lactating sows with transmissible gastroenteritis virus following contact exposure to infected piglets. Cornell Vet 65:352–362. 166796

[118] Wesley RD, Woods RD, Cheung AK. 1991. Genetic analysis of porcine respiratory coronavirus, an attenuated variant of transmissible gastroenteritis virus. J Virol 65:3369–3373. doi:10.1128/JVI.65.6.3369-3373.1991 1851885 PMC240999

[119] Kim L, Hayes J, Lewis P, Parwani AV, Chang KO, Saif LJ. 2000. Molecular characterization and pathogenesis of transmissible gastroenteritis coronavirus (TGEV) and porcine respiratory coronavirus (PRCV) field isolates co-circulating in a swine herd. Arch Virol 145:1133–1147. doi:10.1007/s007050070114 10948987 PMC7086746

[120] Cox E, Pensaert MB, Callebaut P, van Deun K. 1990. Intestinal replication of a porcine respiratory coronavirus closely related antigenically to the enteric transmissible gastroenteritis virus. Vet Microbiol 23:237–243. doi:10.1016/0378-1135(90)90154-n 2169676 PMC7117313

[121] Pensaert M, Callebaut P, Vergote J. 1986. Isolation of a porcine respiratory, non‐enteric coronavirus related to transmissible gastroenteritis. Vet Q 8:257–261. doi:10.1080/01652176.1986.9694050 3018993

[122] Krempl C, Schultze B, Laude H, Herrler G. 1997. Point mutations in the S protein connect the sialic acid binding activity with the enteropathogenicity of transmissible gastroenteritis coronavirus. J Virol 71:3285–3287. doi:10.1128/JVI.71.4.3285-3287.1997 9060696 PMC191465

[123] Chen F, Knutson TP, Rossow S, Saif LJ, Marthaler DG. 2019. Decline of transmissible gastroenteritis virus and its complex evolutionary relationship with porcine respiratory coronavirus in the United States. Sci Rep 9:3953. doi:10.1038/s41598-019-40564-z 30850666 PMC6408454

[124] Song D, Park B. 2012. Porcine epidemic diarrhoea virus: a comprehensive review of molecular epidemiology, diagnosis, and vaccines. Virus Genes 44:167–175. doi:10.1007/s11262-012-0713-1 22270324 PMC7089188

[125] Lee D-K, Park C-K, Kim S-H, Lee C. 2010. Heterogeneity in spike protein genes of porcine epidemic diarrhea viruses isolated in Korea. Virus Res 149:175–182. doi:10.1016/j.virusres.2010.01.015 20132850 PMC7114470

[126] Wang J, Zhao P, Guo L, Liu Y, Du Y, Ren S, Li J, Zhang Y, Fan Y, Huang B, Liu S, Wu J. 2013. Porcine epidemic diarrhea virus variants with high pathogenicity, China. Emerg Infect Dis 19:2048–2049. doi:10.3201/eid1912.121088 24274832 PMC3840889

[127] Li W, Li H, Liu Y, Pan Y, Deng F, Song Y, Tang X, He Q. 2012. New variants of porcine epidemic diarrhea virus, China, 2011. Emerg Infect Dis 18:1350–1353. doi:10.3201/eid1808.120002 22840964 PMC3414035

[128] Diep NV, Norimine J, Sueyoshi M, Lan NT, Yamaguchi R. 2017. Novel porcine epidemic diarrhea virus (PEDV) variants with large deletions in the spike (S) gene coexist with PEDV strains possessing an intact s gene in domestic pigs in Japan: a new disease situation. PLoS One 12:e0170126. doi:10.1371/journal.pone.0170126 28095455 PMC5241010

[129] Oka T, Saif LJ, Marthaler D, Esseili MA, Meulia T, Lin C-M, Vlasova AN, Jung K, Zhang Y, Wang Q. 2014. Cell culture isolation and sequence analysis of genetically diverse US porcine epidemic diarrhea virus strains including a novel strain with a large deletion in the spike gene. Vet Microbiol 173:258–269. doi:10.1016/j.vetmic.2014.08.012 25217400 PMC7126216

[130] Park S, Kim S, Song D, Park B. 2014. Novel porcine epidemic diarrhea virus variant with large genomic deletion, South Korea. Emerg Infect Dis 20:2089–2092. doi:10.3201/eid2012.131642 25424875 PMC4257805

[131] Masuda T, Murakami S, Takahashi O, Miyazaki A, Ohashi S, Yamasato H, Suzuki T. 2015. New porcine epidemic diarrhoea virus variant with a large deletion in the spike gene identified in domestic pigs. Arch Virol 160:2565–2568. doi:10.1007/s00705-015-2522-z 26162305 PMC7087250

[132] Zhang J, Yim-Im W, Chen Q, Zheng Y, Schumacher L, Huang H, Gauger P, Harmon K, Li G. 2018. Identification of porcine epidemic diarrhea virus variant with a large spike gene deletion from a clinical swine sample in the United States. Virus Genes 54:323–327. doi:10.1007/s11262-018-1542-7 29468451 PMC7088737

[133] Su Y, Hou Y, Prarat M, Zhang Y, Wang Q. 2018. New variants of porcine epidemic diarrhea virus with large deletions in the spike protein, identified in the United States, 2016-2017. Arch Virol 163:2485–2489. doi:10.1007/s00705-018-3874-y 29789941 PMC7087112

[134] Su Y, Hou Y, Wang Q. 2019. The enhanced replication of an S-intact PEDV during coinfection with an S1 NTD-del PEDV in piglets. Vet Microbiol 228:202–212. doi:10.1016/j.vetmic.2018.11.025 30593369 PMC7117446

[135] Hou Y, Lin C-M, Yokoyama M, Yount BL, Marthaler D, Douglas AL, Ghimire S, Qin Y, Baric RS, Saif LJ, Wang Q. 2017. Deletion of a 197-amino-acid region in the N-terminal domain of spike protein attenuates porcine epidemic diarrhea virus in piglets. J Virol 91:e00227-17. doi:10.1128/JVI.00227-17 28490591 PMC5487580

[136] Terada Y, Shiozaki Y, Shimoda H, Mahmoud HYAH, Noguchi K, Nagao Y, Shimojima M, Iwata H, Mizuno T, Okuda M, Morimoto M, Hayashi T, Tanaka Y, Mochizuki M, Maeda K. 2012. Feline infectious peritonitis virus with a large deletion in the 5′-terminal region of the spike gene retains its virulence for cats. J Gen Virol 93:1930–1934. doi:10.1099/vir.0.043992-0 22718568

[137] Hulswit RJG, de Haan CAM, Bosch B-J. 2016. Coronavirus spike protein and tropism changes. Adv Virus Res 96:29–57. doi:10.1016/bs.aivir.2016.08.004 27712627 PMC7112277

[138] Corman VM, Baldwin HJ, Tateno AF, Zerbinati RM, Annan A, Owusu M, Nkrumah EE, Maganga GD, Oppong S, Adu-Sarkodie Y, Vallo P, da Silva Filho L, Leroy EM, Thiel V, van der Hoek L, Poon LLM, Tschapka M, Drosten C, Drexler JF. 2015. Evidence for an ancestral association of human coronavirus 229E with bats. J Virol 89:11858–11870. doi:10.1128/JVI.01755-15 26378164 PMC4645311

[139] Wills SE, Beaufrère HH, Brisson BA, Fraser RS, Smith DA. 2018. Pancreatitis and systemic coronavirus infection in a ferret (Mustela putorius furo). Comp Med 68:208–211. doi:10.30802/AALAS-CM-17-000109 29776456 PMC6008714

[140] Ernandes MA, Cantoni AM, Armando F, Corradi A, Ressel L, Tamborini A. 2019. Feline coronavirus-associated myocarditis in a domestic longhair cat. JFMS Open Rep 5:2055116919879256. doi:10.1177/2055116919879256 31636915 PMC6787879

[141] Stephenson N, Swift P, Moeller RB, Worth SJ, Foley J. 2013. Feline infectious peritonitis in a mountain lion (Puma concolor), California, USA. J Wildl Dis 49:408–412. doi:10.7589/2012-08-210 23568918

[142] Slaviero M, Cony FG, da Silva RC, De Lorenzo C, de Almeida BA, Bertolini M, Driemeier D, Pavarini SP, Sonne L. 2024. Pathological findings and patterns of feline infectious peritonitis in the respiratory tract of cats. J Comp Pathol 210:15–24. doi:10.1016/j.jcpa.2024.02.001 38479335

[143] Stout AE, Andre NM, Tejada M, DeTar L, Berliner EA, Whittaker GR. 2021. Identification of pathogens in respiratory samples of shelter cats in New York State. Res Square. doi:10.21203/rs.3.rs-1136156/v1

[144] Griffin DE. 2022. Why does viral RNA sometimes persist after recovery from acute infections? PLoS Biol 20:e3001687. doi:10.1371/journal.pbio.3001687 35648781 PMC9191737

[145] Wells HL, Bonavita CM, Navarrete-Macias I, Vilchez B, Rasmussen AL, Anthony SJ. 2023. The coronavirus recombination pathway. Cell Host Microbe 31:874–889. doi:10.1016/j.chom.2023.05.003 37321171 PMC10265781

[146] Le Poder S. 2011. Feline and canine coronaviruses: common genetic and pathobiological features. Adv Virol 2011:609465. doi:10.1155/2011/609465 22312347 PMC3265309

